# Mapping Vaccine Names in Clinical Trials to Vaccine Ontology using Cascaded Fine-Tuned Domain-Specific Language Models

**DOI:** 10.21203/rs.3.rs-3362256/v1

**Published:** 2023-09-27

**Authors:** Jianfu Li, Yiming Li, Yuanyi Pan, Jinjing Guo, Zenan Sun, Fang Li, Yongqun He, Cui Tao

**Affiliations:** The University of Texas Health Science Center at Houston; The University of Texas Health Science Center at Houston; University of Michigan Medical School; University of Michigan Medical School; The University of Texas Health Science Center at Houston; The University of Texas Health Science Center at Houston; University of Michigan Medical School; The University of Texas Health Science Center at Houston

**Keywords:** Vaccine Ontology, Clinical Trials, Normalization, Domain-specific Language Models

## Abstract

**Background:**

Vaccines have revolutionized public health by providing protection against infectious diseases. They stimulate the immune system and generate memory cells to defend against targeted diseases. Clinical trials evaluate vaccine performance, including dosage, administration routes, and potential side effects. ClinicalTrials.gov is a valuable repository of clinical trial information, but the vaccine data in them lacks standardization, leading to challenges in automatic concept mapping, vaccine-related knowledge development, evidence-based decision-making, and vaccine surveillance.

**Results:**

In this study, we developed a cascaded framework that capitalized on multiple domain knowledge sources, including clinical trials, Unified Medical Language System (UMLS), and the Vaccine Ontology (VO), to enhance the performance of domain-specific language models for automated mapping of VO from clinical trials. The Vaccine Ontology (VO) is a community-based ontology that was developed to promote vaccine data standardization, integration, and computer-assisted reasoning. Our methodology involved extracting and annotating data from various sources. We then performed pre-training on the PubMedBERT model, leading to the development of CTPubMedBERT. Subsequently, we enhanced CTPubMedBERT by incorporating SAPBERT, which was pretrained using the UMLS, resulting in CTPubMedBERT + SAPBERT. Further refinement was accomplished through fine-tuning using the Vaccine Ontology corpus and vaccine data from clinical trials, yielding the CTPubMedBERT + SAPBERT + VO model. Finally, we utilized a collection of pre-trained models, along with the weighted rule-based ensemble approach, to normalize the vaccine corpus and improve the accuracy of the process. The ranking process in concept normalization involves prioritizing and ordering potential concepts to identify the most suitable match for a given context. We conducted a ranking of the Top 10 concepts, and our experimental results demonstrate that our proposed cascaded framework consistently outperformed existing effective baselines on vaccine mapping, achieving 71.8% on top 1 candidate’s accuracy and 90.0% on top 10 candidate’s accuracy.

**Conclusion:**

This study provides a detailed insight into a cascaded framework of fine-tuned domain-specific language models improving mapping of VO from clinical trials. By effectively leveraging domain-specific information and applying weighted rule-based ensembles of different pre-trained BERT models, our framework can significantly enhance the mapping of VO from clinical trials.

## Background

Vaccines have long been widely recognized as one of the significant public health breakthroughs in the past century [1]. By introducing antigens from a pathogen into the human body, vaccines stimulate the immune system to trigger an immune response that leads to the production of memory cells, providing protection against targeted disease and its potential complications [2]. Currently, licensed vaccines are available for more than 30 different infectious diseases, and some of them combined into a single vaccine or administered at a single vaccination encounter [3], [4]. Due to their ability to improve immunity, vaccines have saved millions of lives globally [3]. To support the advancement of vaccine research, development, and implementation, the Vaccine Ontology (VO) [5] has been developed as a community-based ontology. The primary aim of the VO is to promote standardization, integration, and computer-assisted reasoning for vaccine-related data [6]. By providing a structured and standardized framework, the VO facilitates the harmonization and interoperability of vaccine information across different data sources and platforms.

Notably, clinical trials are crucial for ensuring the safety and efficacy of vaccines, playing an essential role in their development by providing critical data [7]. These trials involve testing the vaccine on human subjects under controlled conditions to evaluate its ability to stimulate the immune system and prevent the infection [8]. Moreover, the resulting data collected from these trials are then carefully analyzed to determine the vaccine’s effectiveness and potential side effects, which are critical factors in obtaining regulatory approval for public use [9]. To test various aspects of vaccine performance, including dosage, administration routes, and possible side effects, these trials are usually conducted in several phases [10], [11]. The rigorous testing of vaccines through clinical trials ensures that vaccines are safe and effective, and their benefits outweigh their potential risks [12]. Therefore, clinical trials are widely regarded as the “cornerstone” of vaccine research and development, providing the evidence needed to support decision-making and public health policies regarding vaccine use [13].

ClinicalTrials.gov is a valuable repository of clinical trial information [14]. As of April 2023, ClinicalTrials.gov contains more than 447,000 clinical trial entries submitted by diverse organizations [15]. The Aggregate Analysis of ClinicalTrials.gov (AACT) is a comprehensive and publicly available database derived from the ClinicalTrials.gov registry [16]. It provides detailed information on clinical trials registered in ClinicalTrials.gov, including study characteristics, participant demographics, interventions, and outcomes [17]. However, the vaccine information stored in the clinical trial database is not standardized with non-uniform vaccine names, abbreviations, and codes, which can result in missed vaccination opportunities, duplicate vaccinations, and inaccurate assessments of vaccine coverage, and further lead to confusion and errors in evidence-based decision-making and vaccine surveillance [14]. Thus, normalization for vaccine named entities is an important step to improve the consistency and clarity of vaccine group names toward supporting interoperation between standard vocabularies and optimizing vaccination practices [18]. However, manually harmonizing the full clinical trial information into the clinical trial database is a time-consuming and resource-intensive task [14][17]. Therefore, there is an urgent need to develop accurate and automatic approaches to standardize and link the vaccine name mentioned in the clinical trial entries to the formal concepts in the existing standard terminologies or vocabularies [14].

In the biomedical domain, the task of linking textual mentions to concepts in standard terminology/ontology is called medical concept normalization (MCN) [20].In the 2019 National NLP Clinical Challenges (n2c2), the n2c2/UMass Track on Clinical Concept Normalization aimed to leverage a portion of the i2b2 2010 dataset for the purpose of normalizing specific named entities. These entities encompassed clinical concepts annotated as medical problems, treatments, and tests in the 2010 i2b2/VA Shared Task [21]. Many commonly-used concept normalization tools in the biomedical field (e.g., MetaMap, Mgrep, Negfinder, Peregrine, and Whatizit) use dictionary-based approaches, where MetaMap splits text into chunking that can be identified as concepts, Peregrine finds concepts by string matching and performs word sense disambiguation [22]–[27]. Although dictionary-based approaches for MCN are effective and efficient, they rely on pre-existing dictionaries or terminologies [28]. For instance, dictionary-based approaches may not include all relevant medical concepts or may not be up-to-date with new developments in medicine [28]. Another limitation lies in that dictionary-based approaches may not account for variations in medical terminology or different ways of expressing the same concept, which can lead to inaccuracies in mapping medical concepts to standardized codes [29]. Additionally, dictionary-based methods do not take contextual information into consideration [30].

Inspired by the promise shown by the machine learning approaches in addressing the limitations of dictionary-based medical concept normalization, several studies have utilized the machine learning or deep learning approaches, which learn from large datasets and associated standardized codes to identify patterns and relationships between textual mentions and medical concepts [31], [32]. Wang et al. focused on normalizing mentions in the MCN corpus for the N2C2 2019 shared task[21], [33]. They developed a rule-based multipass sieve approach using dictionaries and achieved an 82.0% accuracy, the highest among rule-based methods [33]. They also experimented with a hybrid method combining the sieve approach and BERT, which achieved a slightly higher accuracy of 82.3% [33]. Pattisapu and his colleagues applied and compared various text embeddings (e.g., AvgEmb, on Bidirectional Encoder Representations from Transformers (BERT), Universal Sentence Encoders (USE), and Embeddings from Language Models (ELMo)) and graph embeddings (DeepWalk, Node2Vec, LINE, and HARP) to encode medical concepts into an embedding space [34]–[42]. Then they trained RoBERTa using stochastic optimizer AdamW [34], [43], [44]. Finally, they used cosine similarity to find the closest medical concept to a given input medical concept mentioned [34]. Miftahutdinov and Tutubalina attempted to map the social media phrase to a relevant medical concept [31]. They solved MCN as a sequence learning problem with robust neural networks like recurrent neural networks as well as developed contextualized word representation models to generate semantic representation of social media posts [31]. Mondal and his colleagues focused on the task of disease linking or normalization, which maps entity mentions in medical text to standard entities in a Knowledge Base (KB) [45]. They proposed a two-phase approach, involving candidate generation and candidate scoring, to rank KB entries based on their similarity to disease mentions [45]. They introduced a robust and portable candidate generation scheme that does not rely on hand-crafted rules, outperforming prior methods by a significant margin on the NCBI disease dataset benchmark [41][42]. Liu and her research fellows developed SAPBERT, a pretraining scheme that tackles the challenge of accurately capturing fine-grained semantic relationships in the biomedical domain [47]. They designed a scalable metric learning framework that aligns the representation space of biomedical entities using Unified Medical Language System (UMLS) [47]. SAPBERT outperformed previous hybrid systems and domain-specific pretrained models, achieving state-of-the-art results in medical entity linking and demonstrating superior performance even without task-specific supervision in the scientific domain [47].

However, there are not many studies related to vaccine concept normalization. Abeysinghe and his research fellows proposed a semi-automated lexical approach to audit vaccine mappings in the Observational Medical Outcomes Partnership (OMOP) vocabulary [48]. They defined mapped and unmapped vaccine pairs, in which mapped vaccine pairs refer to vaccine concepts with a “Map to” relationship; conversely, unmapped vaccine pairs indicate those without a “Map to” relationship [48]. They derived term-difference pairs (e.g., name difference) for mapped and unmapped vaccine pairs based on the representation of each vaccine concept [48]. It would be recognized as a potential mapping inconsistency stemming from the same term-difference pair obtained by both mapped and unmapped vaccine pairs [48]. Miftahutdinov and his colleagues introduced a two-stage neural approach for MCN of diseases and drugs, which originates from BERT [14]. In the training stage, they optimized the relative similarity of mentions and concept names from the ontology by triplet loss, whereas the closest concept name representation in a common embedding space to a given mention representation is obtained in the inference stage [14]. However, their model is dependent on concept names in the terminology used at the inference stage, and additionally, the model does not take into account parent-child relations inherent to the biomedical lexicon [14]. Nonetheless, there still has been no research conducted on standardizing the names of vaccines from clinical trials to align with the VO.

Therefore, the objective of this study is to map VO ontology from vaccine names extracted from clinical trials. The ranking process in concept normalization involves prioritizing and ordering potential concepts to identify the most suitable match for a given context, aiding accurate information retrieval and classification.We developed a cascaded framework that utilized various sources of domain knowledge, including clinical trials, UMLS, and VO, to improve the performance of domain-specific language models for automated mapping of Vaccine Ontology from clinical trials. We conducted a ranking of the Top 10 concepts. The experimental results consistently demonstrated that our proposed cascaded framework outperformed existing baselines in terms of vaccine mapping, achieving an accuracy of 71.8% for the top 1 candidate and 90.0% for the top 10 candidates.

This paper is organized as follows. The overview of the study, the introduction to the datasets and the description of the proposed model, and the experiment setup are included in the Method section. Experiment results are included in the Results section. Discussion and Limitation section covers the discussion of our results, error analysis, and limitations of our study. Last but not least, we summarized our contributions and suggested the directions for future study in the Conclusion section.

## Methods

### Project design and workflow

**Figure 1:**
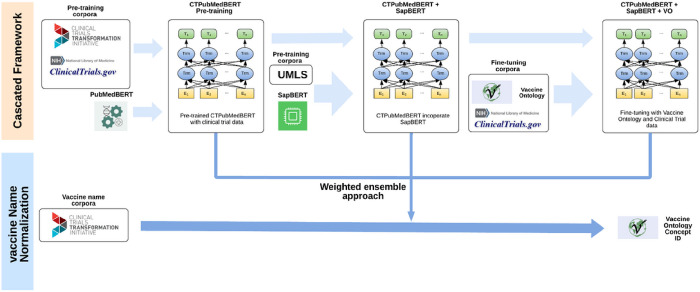
Overview of the cascaded framework.

Figure 1 illustrates an overview of the proposed cascaded framework. Our study aimed to enhance the performance of domain-specific language models for automated mapping of Vaccine Ontology from clinical trials by leveraging multiple knowledge sources, including clinical trials, UMLS, and the VO. Our methodology commenced with the extraction and annotation of data from diverse sources. Subsequently, we conducted pre-training on the PubMedBERT model, resulting in the development of CTPubMedBERT. To further improve CTPubMedBERT, we incorporated SAPBERT, leading to the creation of CTPubMedBERT + SAPBERT. Through fine-tuning using the VO corpus and vaccine data from clinical trials, we achieved further refinement.

### Dataset & data extraction and processing

We obtained the data for our study from three domain knowledge sources: VO [5], UMLS [49], and the AACT database [50].

We downloaded the VO ontology [5] directly from the official website at http://purl.obolibrary.org/obo/vo.owl. This ontology was to establish a standardized vocabulary and collection of concepts that describe the different components, properties, and interactions of different vaccines. It includes both vaccine and non-vaccine terms. To create a reference vocabulary specifically for mapping purposes, we manually filtered out the non-vaccine names from the VO ontology, ensuring that only vaccine-related terms remained.

Clinical trial data was extracted from the AACT database. We downloaded “20230109_clinical_trials.zip” from the Aggregate Analysis of ClinicalTrials.gov (AACT) Database [51]. The downloaded dataset can be accessed through the open source database system like PostgreSQL [52]. The intervention table (named “interventions”) consists of 745,137 record items which contain the vaccine names we intend to obtain. Due to the absence of specific flags in clinical trials to distinguish interventions as vaccines, we employed two string-matching queries to extract vaccine names. In the initial query, we searched for each vaccine term in VO and gathered vaccine names from the interventions that contained the respective vaccine term, using query (1). To ensure uniqueness when a single intervention was matched to multiple VO terms, we applied a TFIDF string-similarity matcher [53]. In the second query, we recognized that some interventions might not directly include the vaccine term but could contain relevant keywords like “vaccine”. To capture such cases, we utilized query (2) to extract additional vaccine names from the intervention table. Subsequently, the results from both queries were combined, and any duplicate names were filtered out. This comprehensive approach aimed to maximize the identification and extraction of vaccine names from the clinical trial data, despite the lack of explicit indications.

SELECT DISTINCT id, nct_id, name FROM ctgov.interventions WHERE intervention_type=‘Biological’ AND (position(lower(‘{vo_label}’) IN lower(name)) > 0 (1)

SELECT DISTINCT id, nct_id, name FROM ctgov.interventions WHERE intervention_type=‘Biological’ AND lower(name) LIKE ‘%vaccine%’ (2)

We downloaded the full release of UMLS-2022AA and prepared the training corpus for fine-tuning language models, according to the approach delineated in [47].

### Annotation

The development of the gold standard involved the participation of two domain experts (J.J. and Y.Y.). From the vaccine names corpus, a total of 550 vaccine terms were selected. Out of these, a random subset of 150 terms was chosen for joint annotation by the experts. They collaborated to annotate these terms and resolved any disagreements through discussions in order to reach a consensus. The Cohen’s kappa agreement between the 2 annotators was 93%.

Following the joint annotation, each expert was assigned 200 terms to annotate independently. Throughout the annotation process, the annotators made every effort to accurately map the vaccine names to the corresponding concepts in the VO. In cases where a direct mapping to a specific concept in the ontology was not possible, the annotators selected the most appropriate concept within the broader category. In the case of conjugate vaccines, the vaccine names with the targeted disease will take higher priority over the superordinate category of the individual vaccines. For example, “MenACWY-CRM conjugate vaccine (Menveo, Novartis)” is mapped to “meningococcal conjugate vaccine”. If neither applied, it’d be assigned to “conjugate vaccine”. On the condition that one vaccine name is mapped to multiple vaccine concepts in VO, each mapping result will be listed. For instance, “23vPPV, dkTpa (Pneumovax, Boostrix)” suggests receiving both vaccines. If the term refers to a vaccine placebo, it will be annotated as the vaccine itself. For example, “AIDSVAX B/E Placebo” will be mapped to “AIDSVAX B/E”. Additionally, if the vaccine term as well as the concept in VO contain both the general name and the product name. The product name will be selected. For example, “2012–2013 trivalent seasonal live attenuated influenza vaccine (FluMist ^®^)” will match the concept “FluMist ^®^”.

### Fine-tuning of Domain-Specific Language Models

PubMedBERT is a domain-specific language model pre-trained on large-scale biomedical corpora [54]. It is commonly used and has achieved state-of-the-art performance in a variety of natural language processing tasks, including biomedical named entity recognition (NER), relation extraction, question answering, and text classification. In this task, we initially pretrained PubMedBERT using Hugging Face Transformers on clinical trials and attempted its use in MCN. By leveraging the developed corpus, we aimed to enhance the model’s understanding of vaccine-related language and concepts. This pre-training process involved exposing the model to a large amount of clinical trial text, allowing it to learn patterns, relationships, and domain-specific knowledge. As a result, the CTPubMedBERT model was developed, equipped with a foundational model in the following cascaded framework.

To further improve the model’s performance, we moved on to re-training the SAPBERT model [55]. SAPBERT is a pre-trained language model based on PubMedBERT that focuses on self-alignment to learn representations of biomedical entities from UMLS. It achieved new state-of-the-art results across six widely used benchmark datasets for biomedical entity linking. This re-training process involved utilizing both the CTPubMedBERT model and the UMLS corpus. By aligning the knowledge from these two sources, we aimed to enhance the model’s understanding of medical terminologies, improving its ability to accurately capture the nuances and context of vaccine-related information. Through this step, the CTPubMedBERT + SAPBERT model was created, incorporating the enhanced capabilities of SAPBERT.

Then, we focused on fine-tuning the CTPubMedBERT + SAPBERT + VO model, leveraging the Vaccine Ontology corpus and vaccine data extracted from clinical trials. This fine-tuning process allowed the model to specifically adapt to the VO and refine its understanding of vaccine-related concepts, classifications, and relationships. By incorporating domain-specific information and aligning it with clinical trial data, the model became more proficient in mapping and analyzing vaccine-related information.

To further enhance the accuracy of the vaccine normalization process, we employed a weighted rule-based ensemble method. This involved combining the predictions of multiple pre-trained models, including CTPubMedBERT + SAPBERT + VO, BIOBERT [56], PubMedBERT, ALL-MPNET [57], SAPBERT, and others. The ensemble method assigned different weights to the top 3 model’s predictions, giving more importance to the models that demonstrated better performance. By aggregating the knowledge and insights from these models, we aimed to achieve higher accuracy and robustness in the normalization of vaccine-related data.

We split our dataset into the training set, validation set, and test set according to the ratio 8:1:1. The model was trained on a server with 8 Nvidia A100 GPU, where each GPU provided a memory capacity of 80GB. The hyperparameters are shown in Table 1.

### Evaluation Procedure

In order to evaluate the vaccine normalization task, we measured the accuracy (Eq. (1)), which quantifies the proportion of correctly predicted concepts relative to the total number of concepts predicted by the system. This metric enabled us to gauge the system’s performance in accurately identifying the correct concept among the suggested options. Furthermore, we assessed the system’s performance by calculating the accuracy at different levels, including Top 1 accuracy, Top 2 accuracy, and so on up to Top 10 accuracy.

(1) $$TopnAccuracy\;=\frac{\left(\sum_{n=1}^{10}\;[\#ofcorrectlypredictedconceptsasTopnsuggestions]\right)}{\left(Total\#ofconceptspredictedbythesystem\right)}$$

## Results

### Results of Data Processing and Screening

**Figure 2:**
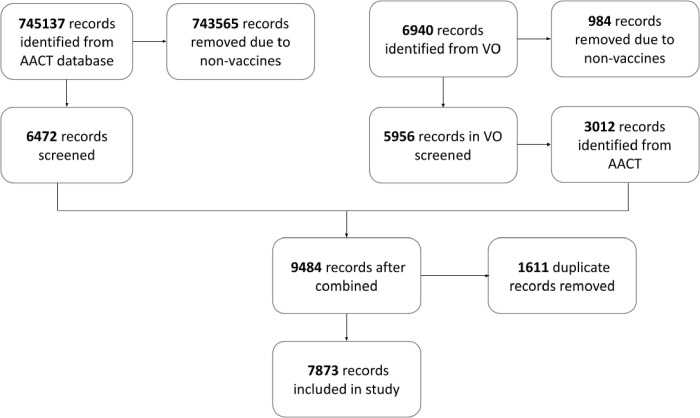
PRISMA flowchart for data extraction and screening with processed results.

Figure 2 shows the process of PRISMA (Preferred Reporting Items for Systematic Reviews and Meta-Analyses) for data extraction and screening with processed results. After two stages of applying string-matching queries, 7873 unique records of vaccine names were extracted from the AACT database. Among them, 550 vaccine terms were selected and annotated by two domain experts for model development and evaluation.

### Results of the mapping performances

Table 2 shows Top 1 to Top 10 accuracy performances from the proposed approach together with a collection of pre-trained language models. It demonstrates that our proposed cascaded framework consistently outperformed existing effective baselines on vaccine mapping, achieving 71.8% on top 1 accuracy and 90.0% on top 10 accuracy.

## Discussion

This study makes multiple contributions. Firstly, we introduce a cascaded framework that utilizes fine-tuned domain-specific language models to map VO terms from vaccine mentions in clinical trials. The framework we propose can seamlessly integrate into existing ontology platforms, enhancing the performance of concept mapping and providing an advanced approach to MCN. Furthermore, we address the issue of ununified granularity in VO terms by enriching and refining the concepts within the ontology. This ensures a more comprehensive and accurate representation of vaccine-related knowledge, improving the overall quality of the ontology.

This task is particularly challenging due to the presence of uncleaned data, variations, and noise in the raw interventions. As demonstrated in the manual annotation procedure, even domain experts may encounter difficulties and confusion when attempting to find certain mapped vaccine concepts. To evaluate the effectiveness of our proposed approach, we conducted a thorough error analysis. This analysis helps us identify the limitations and areas for improvement in our cascaded framework. Additionally, we assess the impacts of employing the cascaded approach and discuss the limitations and future work in detail, which will be elaborated upon below.

### Error Analysis

We categorized the errors into the following major types: NER, abbreviation, disambiguation, hierarchy, semantic, stemming, spelling, and out-of-vocabulary (OOV). Within NER errors, we identified multiple concepts and mentions with noise, while hierarchy was further categorized into ancestor-descendant, parent-child, and sibling based on the hierarchical relations between the gold concepts and the predicted concepts. The descriptions for the error types are shown in Supplement Table 1.

**Figure 3:**
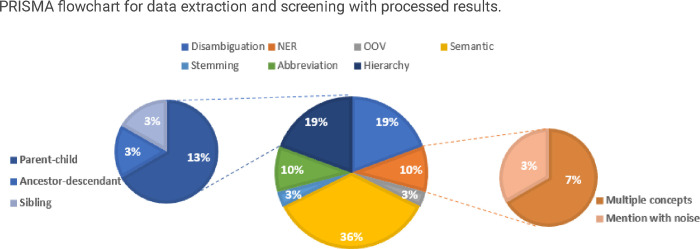
Error types of top-ranked concepts in concept normalization.

The error analysis was conducted on the top-ranked predicted concepts generated by the proposed approach. Figure 3 presents a summarized pie chart of major error types. Out of all the 110 mentions in the test set, 31 mentions failed to map to the correct concept in the top-ranked normalized concept. Interestingly, among the 31 mentions, 19 (61) cases found the correct concept among the Top 10 rankings, emphasizing the notable effectiveness of the proposed approach in concept normalization. The majority of errors (36%) were attributed to semantic errors, with the predicted concept and the gold concept spanning more than two levels. One possible reason for the predicted concept and the correct concept spanning over two levels is the presence of intermediate concepts or subcategories that exist between the two levels. These intermediate concepts can introduce ambiguity or confusion in the mapping process, leading to a mismatch between the predicted concept and the correct concept in terms of their hierarchical placement.

Disambiguation and hierarchy are responsible for the second largest sources of errors, accounting for 19% of the total errors. Disambiguation errors arise when the correct concept is not identified among multiple possible candidate concepts. Multiple factors can account for the presence of multiple concepts within a mention, as well as the disparity between the gold concept and the predicted concept.Firstly, insufficient contextual information may limit the model’s ability to determine the correct concept accurately. Additionally, the concept normalization process may suffer from limited coverage in VO’s vocabulary, potentially leading to inadequate representation of the gold concept, which in turn hinders its accurate prediction. Furthermore, biases or limitations in the training data can influence the model’s ability to predict the gold concept in cases involving multiple concepts. Lastly, inherent limitations of the concept normalization model, such as difficulties in capturing complex relationships or handling multiple concepts, can result in the deviation between the gold and predicted concepts.

In terms of the hierarchy errors, parent-child relationships between the gold concept and the predicted concept accounted for 13% of the total errors, sibling relationships contributed to 3% of the errors, and ancestor-descendant relationships were responsible for 3% of the errors. Several causes can contribute to the occurrence of these hierarchy errors in concept normalization. Firstly, the model may struggle to capture the precise hierarchical relationships between concepts, leading to errors in determining the correct hierarchical placement of concepts. In some cases, the model might mistakenly assign a concept as a parent or child when it should be in a different relationship. Additionally, the limited contextual information or ambiguous mention can make it challenging for the model to accurately identify the exact hierarchical position of the concepts. Moreover, inconsistencies or biases in the training data can also contribute to incorrect hierarchical relationships.

The next largest types of errors in concept normalization were abbreviation errors and NER errors, each accounting for 10% of the total errors. Abbreviations can introduce ambiguity, posing a challenge for the model to accurately map them to the correct expanded concepts. NER errors, on the other hand, mentions containing multiple gold concepts were responsible for 7% of the total errors. Additionally, 3% of the errors were attributed to mentions with noise, further highlighting the need for additional steps in the tokenization process or improvements in the NER task.

Moreover, stemming errors and OOV errors each contribute 3% to the total errors. OOV errors are primarily caused by the incomprehensiveness of the ontology used in concept normalization. For instance, “ad6nsmut mva nsmut” did not map to any concept in VO. Several factors may account for the stemming errors. One reason is the presence of complex or domain-specific terminology that is not adequately handled by the tokenization algorithm, leading to incorrect splitting or merging of words during tokenization. Additionally, non-standard or unconventional language, such as abbreviations, acronyms, or slang, may not be properly recognized or segmented by the tokenization process, resulting in stemming errors. Furthermore, linguistic challenges like compound words, hyphenated words, or words with apostrophes can pose difficulties for accurate stemming. Addressing these issues often requires improving the tokenization and stemming algorithms, incorporating domain-specific knowledge, and developing strategies to handle complex language patterns effectively.

### Impact of Fine-Tuned Domain-Specific Language Models

One of the fundamental elements within our cascaded framework is the incorporation of fine-tuned domain-specific language models. To achieve this, we initially performed pre-training on the PubMedBERT model and further developed CTPubMedBERT by utilizing a clinical trials corpus as the foundational model. Subsequently, we conducted re-training on CTPubMedBERT + SAPBERT using the UML2022A corpus. Finally, we fine-tuned CTPubMedBERT + SAPBERT + VO using the VO ontology.

Our experimental findings demonstrated a significant improvement in the accuracy of the mapping process through the integration of these domain-specific language models. Specifically, there was a notable increase of 16.3% in Top 1 accuracy and 10.9% in Top 10 accuracy compared to the baseline SAPBERT model, which was pre-trained on PubMedBERT (Acc@1: 45.5%, Acc@10: 70.9%). This improvement was observed in the sequentially fine-tuned CTPubMedBERT + SAPBERT + VO model (Acc@1: 61.8%, Acc@10: 81.8%). These results underscore the effectiveness of leveraging fine-tuned language models with domain-specific knowledge to enhance the quality of the normalized outputs.

### Impact of Weighted Rule-based Ensembles

In addition to utilizing fine-tuned language models with multiple domain-specific knowledge, we employed a weighted rule-based ensemble approach to further enhance the normalization of the vaccine corpus. Initially, three ensemble metrics were employed: raw similarity score (Ensemble + Score), scaled similarity score (Ensemble + Scale), and ranking score (Ensemble + Ranking). These metrics were used to assess the performance of the ensemble models. Subsequently, we applied string-matching rules (SM-Rule) to update the normalization by incorporating VO terms if they were present within the interventions.

The weighted rule-based ensembles resulted in a significant enhancement in accuracy, with a 10.0% increase in Top 1 accuracy and an 8.2% increase in Top 10 accuracy compared to the best-performing fine-tuned model, CTPubMedBERT + SAPBERT + VO (Acc@1: 61.8%, Acc@10: 81.8%). Specifically, the weighted scaled-score rule-based ensemble method Ensemble + Scale + SM-Rule achieved an accuracy of 71.8% at Top 1 and 90.9% at Top 10. The progress achieved through the implementation of the string-matching rule effectively addressed the primary obstacles encountered in the VO normalization task, specifically the variability and noise found in vaccine names extracted from interventions in clinical trials. Consequently, this led to a substantial improvement in the quality of the normalization results.

### Limitation and Future Work

However, there are several limitations to consider. Firstly, we rely solely on data from clinical trials, and it is essential to explore data from other sources to test the generalizability of our method. Secondly, the availability of a large amount of annotated data is limited, which can restrict the model’s performance.

In future studies, we aim to improve the validation of vaccine names, both in VO vocabulary and clinical trials. Initially, we performed manual checks to filter out non-vaccine names from VO ontology. However, Ontobee offers extensive support for ontology term dereferencing, linkage, querying, and integration [58]. Vaccine names can be identified from Ontobee using SPARQL queries based on concept tags [58], [59]. To optimize the validation process, we will employ the SPARQL script provided in Supplement Box 1 to extract all vaccine names. What’s more, we plan to introduce a NER step to remove noise from the original data in clinical trials to further improve the performance of the concept normalization task.

## Conclusions

In this paper, we presented a cascaded framework to automatically normalize the vaccine terms in clinical trials based on VO. This includes pre-training of CTPubMedBERT, re-training of SAPBERT, fine-tuning of CTPubMedBERT + SAPBERT + VO, and the utilization of weighted rule-based ensembles. Through this systematic approach, we successfully harnessed fine-tuned domain-specific language models to improve the automated mapping of Vaccine Ontology from clinical trials. Moreover, we supplemented the concepts out of coverage in current VO through our research findings in order to enrich its vocabulary and further enhance its interoperability with other ontologies.

## Figures and Tables

**Table 1:** Hyperparameters of fine-tuning of CTPubMedBERT + SAPBERT + VO.

Hyperparameters	Value
fine-tuning epochs	1
train batch size	256
learning rate	2e-5
max_seq_length of BERT tokenizer	25

**Table 2.** Performances of mapping VO from clinical trials using our proposed approach and other BERT-based models. Acc@n means Top n accuracy, n=1, 2, …, 10

Models	Acc@1	Acc@2	Acc@3	Acc@4	Acc@5	Acc@6	Acc@7	Acc@8	Acc@9	Acc@10

BioBERT- v1.1 [56]	18.2	20.9	22.7	23.6	24.5	24.5	24.5	25.5	25.5	27.3
PubMedBERT [54]	20.9	20.9	23.6	27.3	27.3	27.3	27.3	27.3	27.3	27.3
SAPBERT(+ PubMedBERT) [47]	45.5	53.6	57.3	61.8	63.6	65.5	66.4	68.2	69.1	70.9
CTPubMedBERT+ SAPBERT	45.5	50.9	55.5	59.1	60.0	63.6	63.6	64.5	65.5	67.3
SAPBERT+Pub MedMedBERT+VO	**58.2**	**67.3**	**70.9**	**72.7**	**74.5**	**77.3**	**79.1**	**80.0**	**80.0**	**80.0**
CTPubMedBERT+ SAPBERT+VO	**61.8**	**71.8**	**74.5**	**74.5**	**75.5**	**78.2**	**78.2**	**80.0**	**80.9**	**81.8**
All-MPNET- base-v2 [57]	41.8	51.8	53.6	53.6	57.3	58.2	59.1	60.0	60.9	62.7
All-MPNET- base-v2-sap-ct-vo	**57.3**	**69.1**	**73.6**	**76.4**	**79.1**	**81.8**	**82.7**	**82.7**	**84.5**	**84.5**
Ensemble+Score	60.9	71.8	74.5	74.5	74.5	77.3	78.2	80.9	81.8	81.8
Ensemble+Score+ SM-Rule	70.9	80.0	82.7	82.7	82.7	83.6	83.6	86.4	86.4	86.4
Ensemble+Ranking	62.7	70.9	74.5	75.5	78.2	79.1	80.0	83.6	86.4	87.3
Ensemble+Ranking +SM-Rule	67.3	78.2	83.6	83.6	84.5	85.5	85.5	87.3	89.1	90.0
Ensemble+Scale	60.9	70.0	75.5	77.3	80.0	83.6	85.5	87.3	88.2	88.2
Ensemble+Scale+ SM-Rule	71.8	80.0	82.7	83.6	84.5	85.5	88.2	89.1	90.0	90.0

## Data Availability

Data and materials are available from the corresponding author upon request.

## Abbreviations

AACT: Aggregate Analysis of ClinicalTrials.gov
BERT: Bidirectional Encoder Representations from Transformers
ELMo: Embeddings from Language Models
KB: Knowledge Base
MCN: Medical concept normalization
NER: Named-entity recognition
OMOP: Observational Medical Outcomes Partnership
PRISMA: Systematic Reviews and Meta-Analyses
UMLS: Unified Medical Language System
USE: Universal Sentence Encoders
VO: Vaccine Ontology
